# Thyrotoxicosis Presenting With Acute Cholecystitis: A Case Report and Literature Review

**DOI:** 10.7759/cureus.20840

**Published:** 2021-12-30

**Authors:** Maab F Elhaj, Hiba Magboul, Ashraf O Ahmed, Sreethish Sasi, Ahmed O Saleh

**Affiliations:** 1 Medical Education/ Internal Medicine, Hamad Medical Corporation, Doha, QAT; 2 Medical Education/Internal Medicine, Hamad Medical Corporation, Doha, QAT; 3 Medical Education/ Internal Medicine, Infectious Disease, Hamad Medical Corporation, Doha, QAT; 4 Medical Education/ Internal Medicine, Endocrine and Diabetes, Hamad Medical Corporation, Doha, QAT

**Keywords:** impending thyroid storm, acute surgical abdomen, graves´disease, acalculous cholecystitis, hyperthyroidism

## Abstract

Hyperthyroidism primarily presents with the symptoms and signs of thyrotoxicosis. However, many cases might present with a precipitating factor that unmasks the hyperthyroid status of the patients. These factors are associated with a stress condition, with infections being the most common factors, diabetic ketoacidosis, acute coronary syndrome, and pulmonary embolism. We present a case of hyperthyroidism masqueraded as acalculous cholecystitis.

## Introduction

Hyperthyroidism is one of the most common endocrine disorders. The typical presentation of thyrotoxicosis would be palpitations, weight loss, sweating, tremors, diarrhea, and irregular menstrual cycle. Patients with thyroid storm might present with heart failure, fever, and altered sensorium in the presence of precipitating factors. The most common precipitating factors are infections (most common cause), diabetic ketoacidosis, pulmonary embolism, iodine intake, and other stressors like pregnancy. Pneumonia is being the most common precipitated factor. However, any other infection might act as a stressor that can unmask thyrotoxicosis [1, 2]. Here we report an unusual presentation of thyrotoxicosis precipitated by acalculous cholecystitis.

## Case presentation

A 31-year-old lady with no past medical or surgical history presented to the hospital with abdominal pain, vomiting, and diarrhea. Upon further questioning, she reported having palpitations, tremors, sweating, anxiety and sleep disturbance, and weight loss of 5 kg over the last few weeks. She also reported diarrhea, hair fall, lower limb swelling, shortness of breath, and regular menstrual cycles. She denied any eye symptoms, and she was not an alcohol consumer or smoker.

Upon physical examination, her vitals showed a heart rate: 98 bpm, blood pressure: 130/76 temperature: 36.7 degrees Celcius, maintaining saturation on room air. Examination revealed mild thyromegaly, thyroid bruits, and proximal muscle weakness. There were no thyroid eye changes, pretibial myxedema, or lower limb edema. She also had a tender right upper quadrant of the abdomen with a positive murphy sign.

**Figure 1:**
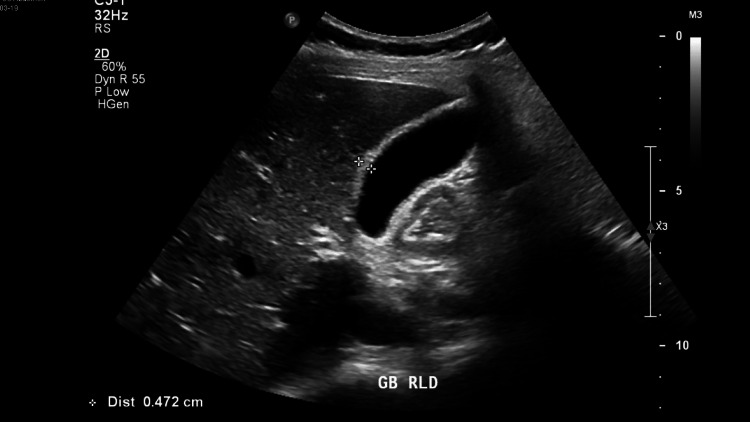
Fig. 1 Gall bladder wall thickening, sludge, and probe tenderness during the examination

Ultrasound abdomen showed biliary sludge and thickened gallbladder wall (Figure 1). She was found to have thyrotoxicosis with free thyroxine (FT4): > 100.0 pmol/L and thyroid-stimulating hormone (TSH): < 0.01 mIU/L. Anti-thyroid stimulating hormone receptor (TSHR) = 16.7. Her general labs showed leukocytosis. Electrolytes, liver enzymes, amylase, and lipase were normal. 

Her Burch-Wartofsky Point scale score was 30 (25-44 supports the diagnosis of thyroid storm). She was managed as a case of thyrotoxicosis with an impending storm and concomitant acalcular cholecystitis. The patient was managed conservatively with antibiotics, intravenous fluids, carbimazole, propranolol, and hydrocortisone (for two days). She had no abdominal pain, nausea, or vomiting. She improved clinically and was discharged home on carbimazole and propranolol after three days.

**Figure 2:**
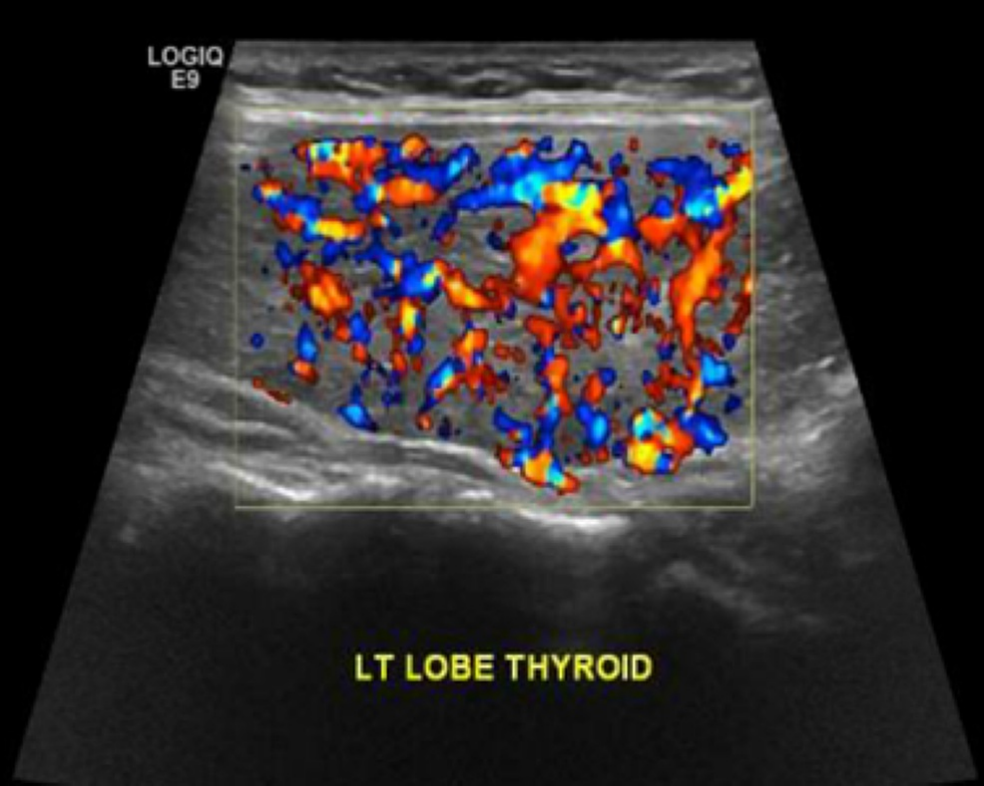
Bulky left thyroid lobe with hypervascularity

**Figure 3:**
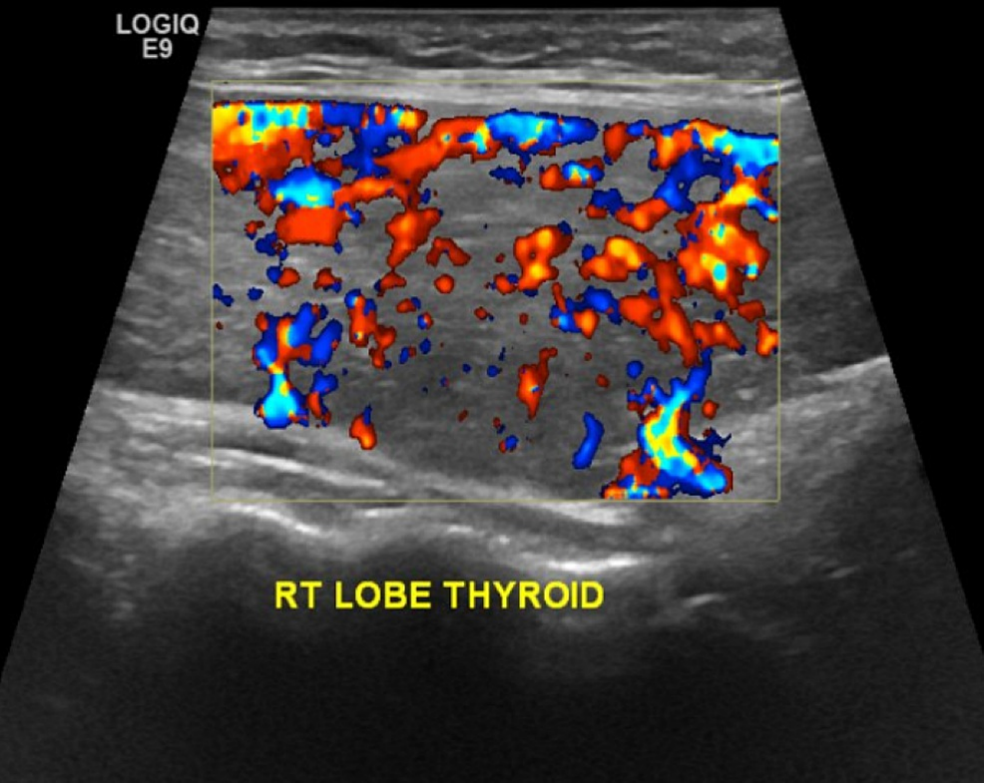
Bulky right thyroid lobe with hypervascularity

Upon follow-up visit in the endocrine clinic, ultrasonography of the thyroid gland showed an enlarged thyroid gland with sonographic features of diffuse thyroid diseases. No sizable cystic or solid nodules were noted (Figures 2, 3). She underwent thyroid radioactive Iodine ablation in October 2021.

## Discussion

Hepatic dysfunctions, including elevated serum transaminases and cholestasis, are usually noted in patients with hyperthyroidism. This relationship was reported more than 100 years ago [1]. Abnormal liver function tests occur in 15-76% of the cases [2]. The hepatic dysfunction can also be secondary to hyperthyroidism complications, including medication adverse effects [3].

Autoimmune liver disease is related to autoimmune thyroid disease, which causes most cases of hyperthyroidism. Evaluation with antinuclear factor, anti-double-stranded deoxyribonucleic acid (DNA), and even liver biopsy is used to differentiate autoimmune hepatitis from other causes of hepatic injury [4, 5]. Hashimoto thyroiditis is thought to be related to primary biliary cirrhosis [4].

Cholestasis may occur in patients with hyperthyroidism. Bile transport is interfered with due to increased hepatic oxygen consumption but without increased hepatic blood flow, thus lowering the oxygen tension in the centrilobular zone [2]. Thyroxine also can cause cholestasis directly [2]. Jaundice from the congestive liver may be secondary to thyrotoxic heart failure [6].

Anti-thyroid agents, including methimazole and propylthiouracil, can cause adverse effects leading to hepatic dysfunction. The injury is likely to be mediated by immune mechanisms. The estimated incidence of anti-thyroid agents associated with hepatotoxicity is about 0.5% [3]. The estimated frequency of immunoallergic hepatitis is 0.1-0.2%. Immunoallergic hepatitis is seen exclusively in patients treated with propylthiouracil. A transient increase in aspartate aminotransferase (AST) and alanine transaminase (ALT) levels is observed in 30% of patients taking propylthiouracil [7]. It happens at all ages and more often in females [2].

Abdominal pain in thyrotoxicosis has simulated a variety of acute surgical emergencies [8]. In this instance, the patient showed typical cholecystitis presentation; thus, arrangements for gall-bladder surgery were instituted [8]. The recognition of thyrotoxicosis led to appropriate treatment and correction of the condition. The patient was treated with propylthiouracil based on probable thyrotoxicosis. Within two weeks, marked improvement was observed [9]. Hepatomegaly resolved, and right upper quadrant tenderness could not be elicited [9]. Treatment with propylthiouracil was continued, with an apparent favorable response. Subtotal thyroidectomy was planned for definitive management of thyrotoxicosis [9]. Moreover, one case has reported ST-segment elevation and acute cholecystitis with uncontrolled hyperthyroidism [10].

Our patient presented with acute cholecystitis and was found to have thyrotoxicosis. Surgery was canceled, was managed conservatively and started on anti-thyroid drugs, and showed marked clinical improvement.

## Conclusions

Our patient presented with acute acalculous cholecystitis that unmasked an impending thyroid storm. However, early recognition and management saved the patient from surgical intervention and thyrotoxicosis complications. It is imperative to recognize the signs and symptoms of thyrotoxicosis in patients presenting with an acute stressor such as an infection. Early recognition and treatment are essential to prevent progression into a thyroid storm and further clinical deterioration.
